# Utility of routine postoperative imaging in adults undergoing primary ventriculoperitoneal shunts

**DOI:** 10.1007/s00701-024-06260-2

**Published:** 2024-10-15

**Authors:** Adnan R. Alnaser, Abed Alnsour, Omar N. Pathmanaban, Helen Maye, Catherine McMahon, Matthew Bailey, Mueez Waqar

**Affiliations:** Department of Neurosurgery, Geoffrey Jefferson Brain Research Centre, Northern Care Alliance NHS Foundation Trust, Manchester, M6 8HD UK

**Keywords:** Ventriculoperitoneal shunt, Hydrocephalus, Imaging

## Abstract

**Background:**

There is currently no consensus on the usefulness of postoperative imaging after ventriculoperitoneal (VP) shunt insertion in adults. The aim of this study was to investigate the utility of routine postoperative imaging (CT head scans and radiographs) following primary VP shunt insertion in a general adult population treated at a tertiary neurosurgical centre.

**Methods:**

Patients undergoing primary VP shunt insertion between 2017–2021 were included. Actions taken based on routine postoperative imaging and need for subsequent shunt revision were recorded.

**Results:**

236 patients were included. The median age was 63 years (range 17–90). There was a slight female preponderance (121/236, 51.3%). The median follow-up was 38.5 months (3.1 – 60.5 months). Acute intervention was employed in 9 patients (3.9%) on the basis of routine postoperative CT head scan. Routine postoperative radiographs did not result in reoperation. Around a quarter (28.8%) of patients had a shunt revision, most of whom underwent urgent primary shunt insertions. Postoperative ventricular catheter characteristics (position of shunt tip, tip relation to septum pellucidum, and intraventricular catheter distance) were not predictive of shunt revision. Surgical urgency (emergency vs. elective procedures) was associated with long-term shunt revision (OR = 2.80, 95% CI 1.42 – 5.53, *p* = 0.003).

**Conclusions:**

Routine postoperative imaging rarely led to reoperation in adult patients undergoing primary VP shunt insertion. Patients undergoing emergency shunt insertions were at the highest risk for requiring revision.

## Introduction

Ventriculoperitoneal shunts (VP shunt) are the most commonly used treatment for adult hydrocephalus [7, 10]. Many neurosurgical departments routinely perform computed tomography (CT) of the head and plain radiographs of the skull/neck, chest, and abdomen (shunt series) in the immediate postoperative period (up to 72 h) following VP shunt insertion to ensure adequate catheter placement and to detect postoperative complications such as haemorrhage, pneumocephalus or shunt over drainage. However, there is no consensus as to whether routine postoperative imaging is warranted after VP shunt insertion or if clinical status alone is sufficient [8].

Ionising radiation has associated potential risks of neoplasia, so this discussion is relevant and warranted, though understudied. In this study we investigated the utility of routine postoperative imaging (CT scan and radiographs) following primary VP shunt insertion in a general adult population treated at a tertiary neurosurgical centre. Utility in this context was to detect early complications and specifically, the primary outcome was to evaluate the percentage of routine postoperative imaging studies that led to an acute intervention.

## Methods

### Data acquisition

This study was approved by the institutional review board. All cases of primary VP shunt insertion between 2017 and 2021 were collected from the health records of a tertiary neurosurgical referral centre. Inclusion criteria included adults (≥ 16 years) undergoing primary VP shunt insertion. Exclusion criteria included revision cases or patients undergoing other types of shunt procedures, and patients with follow up duration less than 3 months.

Data were collected on demographics, aetiology of hydrocephalus, operative details of shunt insertion (including urgency) and routine postoperative imaging. Elective shunts were categorised as non-urgent and those performed on our neurosurgical emergency list were categorised as urgent. Image guidance was defined as stereotactic shunt insertion.

### Imaging review

Postoperative imaging usually comprises a CT head scan and shunt series (X-ray skull, chest, and abdomen). Routine postoperative imaging was defined as studies performed within 72 h postoperatively in the absence of any symptoms or indicators of pathology. Actions taken based on routine postoperative imaging and need for subsequent shunt revision were recorded. Postoperative ventricular catheter characteristics (position of shunt tip, tip relation to septum pellucidum, and intraventricular catheter distance) were assessed using CT images and radiological reports.

The primary outcome was the percentage of routine postoperative imaging studies that led to an acute intervention. Secondary outcomes included future shunt revision.

### Statistics

SPSS version 25 was used for data analysis. Univariate analysis using Chi-squared and Fisher Exact tests were used to compare proportions. Multivariate analysis was performed using a logistic regression model, including variables were *p* < 0.15 at the univariate level. Shunt survival curves were plotted using Kaplan–Meier plots and comparisons were made using the Log-rank test.

## Results

### Patient characteristics

A total of 236 patients were included (10 excluded due to short follow up duration – less than 3 months). The median age was 63 years (range 17–90). There was a female preponderance (121/236, 51.3%). Brain tumour associated hydrocephalus (77/236, 32.6%) and Normal Pressure Hydrocephalus (NPH) (71/236, 30.1%) were the most common aetiologies of hydrocephalus.

**Table 1:** Patient characteristics (*n* = 236)

	Absolute count (median/%)
Age:	236 (63 years)
Age groups:	
52 years or less	80 (33.9)
52 – 71 years	80 (33.9)
+ 71 years	76 (32.2)
Gender:	
Male	115 (48.7)
Female	121 (51.3)
Cause of hydrocephalus:	
Tumour	77 (32.6)
NPH	71 (30.1)
Vascular	38 (16.2)
Congenital	21 (8.9)
IIH	13 (5.5)
Trauma	12 (5.1)
Infection	4 (1.7)
Urgency:	
Elective	104 (44.1)
Emergency	132 (55.9)
Performing surgeon:	
Consultant present	173 (70.3)
Non consultant	73 (29.7)
Imaging requested and performed:	
Yes	69 (29.2.9)
No	167 (70.8.1)
Imaging (brainlab/stealth reported in op notes):	
Yes	62 (26.3)
No	174 (73.7)
US guidance used:	
Yes	158 (66.9)
No	78 (33.1)
Shunt valve type:	
Codman Hakim	143 (60.6)
Certas	39 (16.5)
Progav	25 (10.6)
OSV-II	22 (9.3)
Fixed pressure	7 (3.0)
Shunt adjunct used:	
Yes	201 (85.2)
No	35 (14.8)
Postoperative imaging: *Skull Xray:*	
Yes	31 (13.1)
No	205 (86.9)
*Chest Xray:*	
Yes	56 (23.7)
No	180 (76.3)
*Abdominal Xray:*	
Yes	128 (54.2)
No	108 (45.8)
*CT head:*	
Yes	227 (96.8)
No	9 (3.8)
Low-dose CT:	
Yes	172 (72.9)
No	64 (27.1)

**Table 2:** Postoperative ventricular catheter characteristics (*n* = 236)

	Absolute count (median/%)
Position of shunt tip:	
No imaging	9 (3.8)
Body of lateral ventricle	166 (70.3)
Trigone	23 (9.7)
Frontal horn	18 (7.8)
Foramen of Monro	5 (2.1)
3rd Ventricle	4 (1.7)
Other parenchymal location	11 (4.7)
Tip relation to septum pellucidum:	
No imaging	9 (3.8)
Touch the septum	91 (38.6)
Cross the septum	56 (23.7)
No touch/cross	80 (33.9)
Mean Intraventricular catheter distance:	33.3 mm
Intraventricular catheter distance groups:	
No imaging	9 (3.8)
Less than 3.5 cm	116 (49.2)
3.5 cm or more	111 (47.0)

There was a roughly equal split between emergency (55.9%) and elective shunt procedures (44.1%). Image guidance was used in 104/236 (44.1%) patients and US guidance was used in 158/236 (66.9%). Further baseline characteristics are shown in Table 1.

Shunt valves utilised included: Codman® Hakim® (60.6%), Codman® Certas® (16.5%), proGAV® II (10.6%) and OSV II® (9.3%).

### Postoperative imaging

**Fig. 1:**
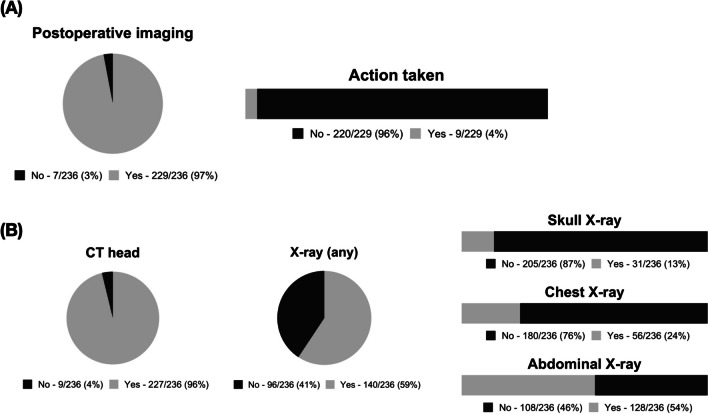
Summary of study findings. (**A**) Proportion of patients who had routine postoperative imaging (97%) and those who had intervention based on this imaging. (**B**) Portion of patients who had a routine postoperative head CT (96.2%), and/or plain radiographs (59.3%)

Almost all patients had at least one routine postoperative imaging study (97%, Fig. 1A), most commonly (96.8%) a CT head scan. Of those, 172/227 (72.9%) had a low dose scan. An abdominal X-ray was performed in 54.2%. Over one third (40.7%) did not have any X-rays postoperatively (Fig. 1B).

Catheter tips were located in the lateral ventricular body (70.3%), trigone (9.7%) or frontal horn (7.8%). Around one third (38.6%) made contact with the septum pellucidum and 56/227 (23.7%) crossed it. Around half (49.2%) of patients had an intraventricular catheter distance of less than 3.5 cm. Postoperative imaging characteristics are summarised in Table 2.

### Actions taken based on postoperative imaging

The vast majority of patients (96.2%) required no action based on postoperative imaging (Fig. 1A). However, intervention was employed in 9 patients out of 229 (3.9%): 4 patients had revision due to unchanged/worsening ventricular size, 4 patients had revision due to catheter malposition - located either in an extraventricular location (3) or in the temporal horn (1), and 1 patient (< 1%) had intraventricular haemorrhage. During the study period, no patients had actions taken on the basis of a skull X-ray, chest X-ray or abdominal X-ray.

We evaluated factors predictive of action based on postoperative imaging (Table 3). Surgical urgency demonstrated a trend towards significance (*p* = 0.08), although other factors including age (*p* = 0.74), gender (*p* = 0.50), image guidance (*p* = 0.70) and USS (*p* =  > 0.99) were not significant.

**Table 3:** Need for action based on routine postoperative imaging (*n* = 236)

	Number of cases with action / total group count (%)	Univariate (Chi-square/Fisher)	*P*-value
Age:		0.60	0.74
52 years or less	3/77 (3.9)		
52 – 71 years	4/76 (5.0)		
+ 71 years	2/74 (2.6)		
Gender:		F	0.50
Male	3/112 (2.6)		
Female	6/115 (5.0)		
Urgency:		F	0.08
Emergency procedure	8/124 (6.1)		
Elective procedure	1/103 (1.0)		
Performing surgeon:		F	0.72
Consultant present	6/161 (3.6)		
Non consultant	3/66 (4.3)		
Cause of hydrocephalus:		4.85	0.56
Tumour	5/72 (6.5)		
NPH	1/70 (1.3)		
Vascular	2/36 (5.3)		
Congenital	0/21 (0.0)		
IIH	1/12 (7.7)		
Trauma	0/12 (0.0)		
Infection	0/4 (0.0)		
Image guided insertion:		F	0.70
Yes	3/59 (4.8)		
No	6/168 (3.4)		
US guided insertion:		F	> 0.99
Yes	6/152 (3.8)		
No	3/75 (3.8)		

*F* = Fisher exact test used

Seven patients did not have routine postoperative imaging during the study period. Of these, only one was an elective case and six were urgent insertions. Only one patient required a shunt revision after 91 days, who originally had an urgent shunt inserted for symptoms consistent with normal pressure hydrocephalus.

### Shunt revisions

The median follow-up was 38.5 months (3.1 – 60.5 months). Around a quarter (28.8%) of patients had a shunt revision after a median time of 60.4 days (range 1–1641). Overall, 12/68 (17.6%) had a revision within one week of the primary procedure.

**Fig. 2:**
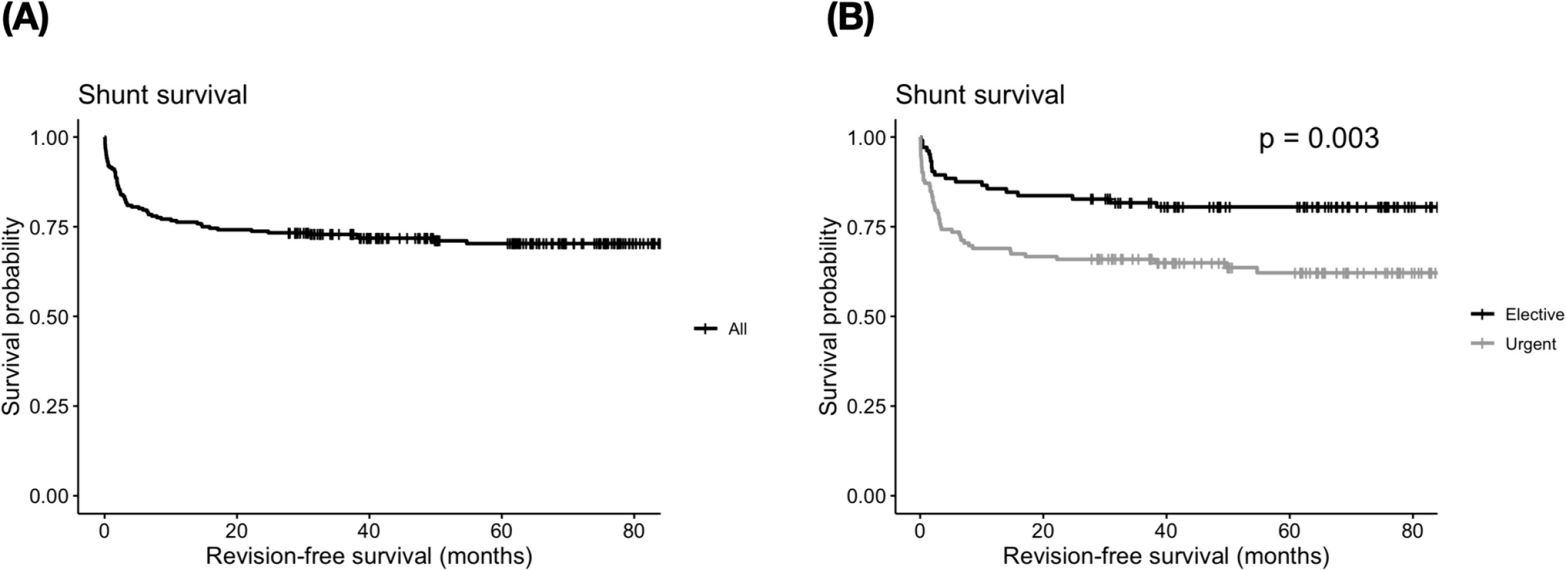
Shunt survival: the effect of procedural urgency. (**A**) Overall study cohort shunt survival curve. (**B**) Urgency of procedure was associated with a significantly shorter time to revision (Log-rank test, *p* = 0.003)

We evaluated factors associated with shunt revision (Table 4). In multivariate analysis, only surgical urgency was associated with the need for a shunt revision (OR = 2.80, 95% CI 1.42 – 5.53, *p* = 0.003). Interestingly, factors such as use of image guidance were not. A shunt survival curve demonstrating the effect of surgical urgency is demonstrated in Fig. 2.

**Table 4:** Shunt revision among patients who have had a postoperative CT scan (*n* = 227)

	Shunt revision cases / total group count (%)	Univariate (Chi-square/Fisher)	*P*-value	Multivariate analysis
Age:		4.2	0.12	OR = 0.86, 95% CI 0.58 – 1.27, *p* = 0.45
52 years or less	27/49 (35.5)			
52 – 71 years	24/54 (30.8)			
+ 71 years	15/58 (20.5)			
Gender:		F	0.77	Not entered
Male	33/77 (30)			
Female	33/84 (28.2.8)			
Urgency:		F	0.002*	OR = 2.80, 95% CI 1.42 – 5.53, *p* = 0.003
Emergency procedure	47/78 (37.6)			
Elective procedure	19/83 (18.6)			
Performing surgeon:		F	0.20	Not entered
Consultant	43/119 (26.50)			
Non consultant	23/42 (35.4)			
Intra-op image guidance used (brainlab/stealth):		F	0.74	Not entered
Yes	18/40 (31.0)			
No	48/121 (28.4)			
Intra-op image guidance used (US):		F	0.35	Not entered
Yes	42/113 (27.1)			
No	24/48 (33.3.3)			
Intra-op use of ANY imaging:		F	0.77	Not entered
Yes	27/71 (27.6)			
No	39/90 (30.2)			
Cranial tip position:		8.4	0.16	Not entered
Body of lateral ventricle	45/121 (27.1)			
Trigone	5/18 (21.7)			
Frontal horn	5/13 (27.8)			
Foramen of Monro	2/3 (40.0)			
3rd Ventricle	2/2 (50.0)			
Other	7/4 (63.6)			
Tip relation to septum pellucidum:		3.84	0.16	Not entered
Touch the septum	20/71 (22.0)			
Cross the septum	18/38 (32.1)			
No touch/cross	28/52 (35.0)			
Intraventricular catheter distance:		F	0.14	OR = 0.62, 95% CI 0.34 – 1.15, *p* = 0.13
Less than 3.5 cm	39/77 (33.6)			
3.5 cm or more	27/84 (24.3)			
Shunt valve type:		3.08	0.54	Not entered
Codman Hakim	41/98 (29.5)			
Certas	10/27 (27.0)			
Progav	4/19 (17.4)			
OSV-II	8/13 (38.1)			
Fixed pressure	3/4 (42.9)			
Shunt adjunct:		F	> 0.99	Not entered
Yes	10/24 (29.4)			
No	56/137 (29.0)			
Cause of hydrocephalus:		14.16	0.028*	OR = 1.20, 95% CI 0.98 – 1.47, *p* = 0.07
Tumour	27/46 (37.0)			
NPH	14/55 (20.3)			
Vascular	8/29 (21.6)			
Congenital	3/17 (15.0)			
IIH	7/6 (53.8)			
Trauma	6/6 (50.0)			
Infection	1/2 (33.3)			
Postoperative AXR:		F	0.88	Not entered
Yes	36/90 (28.6)			
No	30/70 (29.7)			

*F*: Fisher exact test used, *AXR*: Abdominal X-ray

## Discussion

This study assessed the utility of routine postoperative imaging in a general adult population of patients undergoing primary elective/emergency VP shunt insertion. Most patients had brain tumour associated, normal pressure hydrocephalus (NPH), or vascular-event related hydrocephalus. Postoperative cranial imaging was nearly always employed but radiographs were less common and useful. Reoperation was rarely warranted based on cranial imaging (3.9%) but more common in urgent cases. Urgency of procedure was also predictive of long-term revision. Postoperative imaging characteristics were not predictive of long-term revision.

Use of routine postoperative imaging should be justified clinically especially when ionising radiation methods are used. After VP shunt placement, postoperative imaging is primarily used to assess the adequacy of ventricular/abdominal catheter position, which can also serve as a baseline for future follow-up imaging. We found a low rate of intervention, and this is consistent with existing data. A German study reported 2.3% (11 out of 479) of routine postoperative CT scans resulted in clinical intervention (revision surgery), while malposition was found in 9 cases and intracerebral haemorrhage was found in 2 cases [2]. A Swiss study reported a yield rate of 1.7% of early postoperative CT scans, where 3 patients underwent revision based solely on imaging [8]. In the paediatric age group, a similarly low yield of 0.8% (3 out 244 cases) has been reported [6]. The overall rate of intervention is therefore low so available data could be used to justify avoiding postoperative cranial imaging routinely, especially where access is limited.

A lower reintervention risk does not necessarily counter the potential benefits of postoperative imaging after shunt insertion, especially when clinical assessment is limited. This may be the case, for example, in patients with reduced baseline GCS after vascular/trauma related hydrocephalus. This may be even more compelling with lower-dose CT protocols, which provide sufficient resolution to assess catheter placement whilst minimising radiation dose [14].

Routine postoperative radiographs did not result in intervention in this study. Other studies have also reported a limited utility of plain radiographs in evaluation of shunt failure [3, 4, 11]. Abnormal radiographs were found in only 1 out of 296 cases (0.3%) who underwent surgical revision in the absence of an abnormal CT head scan [11]. This investigation could therefore be avoided after shunt placement unless there is clinical concern (e.g. difficult intra-operative access, postoperative swelling).

Urgency of shunt insertion was predictive of future revision in our study. At our centre, urgent shunts are usually inserted in a dedicated neurosurgical emergency theatre and by neurosurgical trainees with/without consultant (attending) supervision. This factor does not explain the higher risk of revision however, as grade of performing surgeon did not correlate with need for revision (Table 4). Our findings correlate with national data from the USA in NPH patients reporting that urgency of shunt insertion was associated with need for shunt revision, prolonged hospital stay and increased economic burden [1]. The underlying reasons for this are unclear and could be multifactorial due to human factors (e.g. time of operating, lack of regular theatre team, time pressures from concurrent emergencies) and a more complex patient group requiring inpatient shunt insertion. Other neurosurgical procedures such as tumour resection also have inferior outcomes when performed on an urgent versus elective basis [13].

Image-guided ventricular catheter placement was associated with comparable shunt failure rates to free hand insertion in our study. This is similar to a recent meta-analysis in paediatric patients undergoing primary VP shunt insertion, in which image-guided insertion did not result in lower revision rates when compared to hand-free insertion [12]. In another study, although stereotactic placement improved accuracy of ventricular catheter placement, it did not influence long-term shunt failure rates [9]. Despite this data however, we still advocate use of image guidance during shunt insertion to minimise the risk of complications.

We did not identify any ventricular catheter characteristics that predicted shunt failure. This is in-keeping with a secondary analysis of 3 large paediatric studies in which catheter tip location was not related to shunt revision [15]. A study from Japan reported contrary results however and tips located in the third ventricle, contacting the ventricular wall, or in the septum pellucidum were associated with shunt malfunction [16]. Conversely, a recent study from Italy also found that tips positioned in the foramen of Monro, third ventricle and lateral ventricles resulted in a lower rate of shunt revision compared to tips positioned in the septum pellucidum or wall of the lateral ventricle [5].

Limitations of the present study include its single-centre retrospective nature. Within the study period although there were no cases of abdominal radiographs requiring intervention, anecdotally in the authors’ experience there have been cases where this investigation has been useful. This includes patients that required revision of their abdominal catheters at a later date, when initial postoperative imaging confirmed intraperitoneal catheter placement.

## Conclusions

In this study, we evaluated the utility of routine postoperative imaging in a general adult population of patients undergoing primary elective/emergency VP shunt insertion. Intervention (reoperation) was rarely warranted based on cranial imaging (3.8%), never based on radiographs (0%) but more common in urgent cases. Urgency of procedure was also predictive of long-term revision. Postoperative imaging characteristics were not predictive of long-term revision. Our data supports a more personalised approach to postoperative imaging after VP shunt insertion, avoiding its use in all cases.

## Abbreviations

VP: Ventriculoperitoneal
US guidance: Ultrasound guidance
CT scan: Computed Tomography scan
NPH: Normal Pressure Hydrocephalus
